# Mapping the landscape of students' creative thinking ability: a systematic literature review and future pathways

**DOI:** 10.3389/fpsyg.2025.1692056

**Published:** 2026-02-11

**Authors:** Maryam Ikram, Buer Song, Ziyang Wang, Ge Tian

**Affiliations:** 1 Faculty of Education and Liberal Arts, INTI International University, Nilai, Negeri Sembilan, Malaysia; 2 Xilingol Vocational College, Xilinhot, China

**Keywords:** cognitive learning theory, creative thinking ability, learning opportunities, meta-literature review, systematic literature review, education quality

## Abstract

**Introduction:**

This study aims to provide researchers with a comprehensive literature review that illustrates how students' creative thinking ability has informed the development of meaningful research pathways.

**Methods:**

The systematic review highlights the significance of incorporating student perspectives and is grounded in empirical studies published between 1954 and 2025, retrieved from the Scopus database. From an initial pool of 186 studies, 30 met the inclusion criteria and were analyzed qualitatively.

**Results:**

The review provides an in-depth portrayal of how creative thinking among students is addressed within educational research. While quantitative methods dominate, they often fall short in capturing the complexity of motivational factors. In contrast, qualitative approaches present opportunities for methodological innovation. The geographical diversity of the studies particularly the strong representation from Asia underscores the global relevance of this topic.

**Discussion:**

This review not only maps current research trends but also identifies promising directions for future inquiry, advocating for flexible and context-sensitive strategies to enhance students' creative thinking. To the best of our knowledge, this systematic review integrates analyses of creative thinking with related meta-literature, addressing an underexplored area and contributing to ongoing academic development.

## 1 Introduction and literature review

Creative thinking is characterized by the ability to introduce unique ideas and products that stand apart from established solutions. While common sense thinking uses rational and methodical approaches to determine accurate conclusions, divergent thinking emphasizes the production of diverse and original ideas when engaging with mathematical tasks such as problem solving and posing. The creative process integrates critical thinking, which seeks fresh perspectives, with vertical thinking, which focuses on elaboration and objective evaluation, ensuring a balanced approach to innovation.

Creativity stems from individuals who possess the capacity for creative thinking (Finney, 2010). This skill is essential not only for the present generation but also for future ones (Gube and Lajoie, 2020; Yang et al., 2022). According to (Yildiz and Guler Yildiz 2021), nurturing creative thinking should begin at an early age to prepare students for upcoming challenges. Creativity enables individuals to generate innovative and original ideas, resulting in unique and valuable outcomes (Abraham, 2016). It is the driving force behind human accomplishments such as space exploration, artistic creation, technological advancement, and medical breakthroughs (Ritter and Mostert, 2017). The quality of education can be assessed not solely through academic performance, but also through students' ability to think independently, produce novel ideas, and demonstrate broad perspectives (Albar and Southcott, 2021).

The United Nations (UNESCO) promotes the slogan “Literacy for All,” advocating for universal access to literacy as an essential asset for future readiness. Literacy is known to have extensive benefits, including combating poverty, lowering infant death rates, regulating population growth, supporting gender parity, and advancing sustainability, peace, and democratic governance (dan Rahmawati Badan Penelitian dan Pengembangan, 2014). In the 2012 PISA assessment draft, mathematical literacy is defined as a person's ability to apply, interpret, and analyze mathematics across various contexts. This includes using mathematical knowledge, concepts, facts, procedures, and tools to make sense of and anticipate real-life situations (Buyung and Dwijanto, 2017). Generally, literacy is associated with the use of language, where written language is viewed as a supporting, rather than primary form. Since language evolves within cultural frameworks, literacy must be defined in a way that reflects its socio-cultural dimensions (dan Rahmawati Badan Penelitian dan Pengembangan, 2014).

Assessing individuals' creative thinking ability is a key aspect of education. One of the most commonly used tools for this purpose is the Torrance Tests of Creative Thinking (TTCT) (Bart et al., 2017). According to Hokanson, the TTCT developed by Torrance and his colleagues is widely recognized, available in 35 languages, and primarily designed to identify gifted children (Kim, 2006). However, the criteria used in TTCT are not well-suited for evaluating mathematical creative thinking, particularly mathematical communication, as this domain is often considered more specialized than general creative thinking (Singer and Voica, 2015).

To measure creative thinking in a broader population including highly creative individuals like artists and scientists researchers have developed psychometric approaches. These approaches often assess divergent thinking, which is considered synonymous with creative thinking. Tools such as Guilford's Alternate Uses test are used to evaluate divergent thinking and can reliably predict creative outcomes (Runco and Acar, 2012).

**Figure 1:**
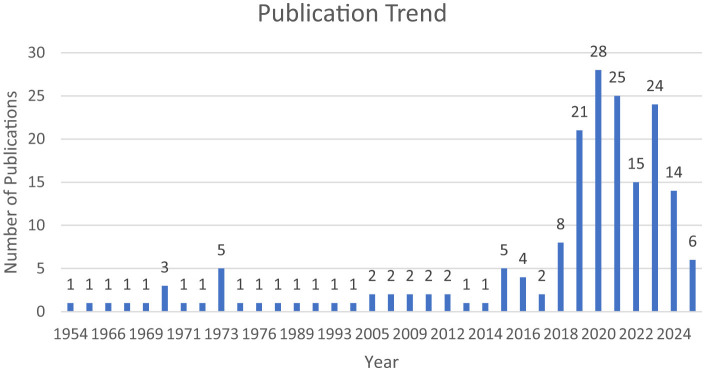
Growth of creative thinking ability publications from 1954-2025. Source: Scopus database.

Despite its educational importance, creative thinking remains an under-explored construct in the previous literature, particularly in relation to foundational models in the social sciences and humanities. Over the past seventy years, research has largely examined creative thinking through isolated variables or within fragmented disciplinary domains, limiting cumulative theoretical development. A preliminary scoping of Scopus-indexed publications from 1954 to July 2025 revealed a notable gap: no existing systematic reviews or meta-analyses have integrated creative thinking within conceptual development or educational psychology frameworks (see Figure 1). This gap underscores the need for a systematic review to consolidate dispersed evidence, clarify conceptual foundations and support theory-building. Accordingly, this systematic review integrates research on creative thinking with related meta literature, offering an integrated perspective that may support future studies and inform educational practice and policy.

This absence of integrated reviews underscores the need for a new SLR that not only consolidates scattered findings but also offers a conceptual foundation for future empirical and theoretical work. By identifying and categorizing research trends, gaps and future directions, this review addresses a critical void in the literature. It strengthens the conceptual framing of creative thinking as a multidimensional psychological construct, intersecting with literacy, cognition, innovation and institutional change.

To fill this scholarly gap, the present review investigates creative thinking ability among students, especially in relation to institutional risks and systemic challenges. While creativity is widely acknowledged as a key 21st-century skill, there remains a lack of research connecting it to broader educational vulnerabilities and strategic decision-making. This study contributes by examining how limitations in creative thinking may adversely affect educational institutions, from curriculum design to sustainability policies. Drawing on prior work and new qualitative synthesis, it provides actionable insights into how creative thinking informs teaching practices, supports educational resilience, and shapes policy development.

## 2 Methodology

This study's methodology is built around two major components: the review protocol and the meta-literature review. The review is carried out in four stages, beginning with the PRISMA protocol, followed by formulating research questions using PICo, applying systematic search methods, and finally conducting integrated data extraction and analytical procedures.

### 2.1 The review protocol – PRISMA

Following the Preferred Reporting Items for Systematic Reviews and Meta-Analyses (PRISMA) guidelines, a structured and transparent review of the relevant literature was conducted (Ikram et al., 2025; Rethlefsen et al., 2021). These standards guide researchers on the essential elements to report, which strengthens the overall assessment of review quality. While PRISMA was originally designed for systematic reviews of randomized trials, it is also applicable to other forms of systematic reviews (Moher et al., 2009). According to (Sierra-Correa and Cantera Kintz 2015), PRISMA offers several benefits, such as establishing clear inclusion and exclusion criteria, sharpening the focus of research topics, and enabling efficient screening of studies across large databases. The PRISMA framework further supports comprehensive searches of terms related to creative thinking ability within Social Science, Psychology, and Arts and Humanities fields, which aligns with the purpose of this study (Page et al., 2021). Therefore, PRISMA is an appropriate methodological choice for examining “creative thinking ability.”

### 2.2 Formulation of research question

The research topic in this study was formulated using the PICo framework. In systematic reviews, PICo serves as a structured tool that supports researchers in shaping an appropriate and focused research topic (Dhir and Gupta, 2021). The framework consists of three elements: Population or Problem, Interest, and Context. In this review, these elements were defined as articles (Population), creative thinking ability (Interest), and the Social Science, Psychology, and Arts and Humanities fields (Context). Together, these components informed the development of the research questions.

### 2.3 Systematic search strategies

The systematic search process consists of three key stages: identification, screening and determining eligibility.

#### 2.3.1 Identification

In this study, a substantial pool of potentially relevant articles was gathered. The selection process was carried out in three steps. The first step involved identifying suitable keywords, followed by searchingThe first step involved identifying suitable keywords, followed by searching for related terms and synonyms based on previous studies and supporting sources (Ikram and Kenayathulla, 2022; Wider et al., 2025). These terms were then used to construct a detailed search query within the Scopus database. Scopus is a widely recognized multidisciplinary indexing platform and is considered appropriate for systematic literature reviews because of its emphasis on article quality (Mehmood et al., 2022). Using the search query applied to article titles, a dataset was generated (Table 1) containing publications that matched the targeted keywords. During the initial phase of the review, a total of 186 articles were retrieved from Scopus.

**Table 1:** The search string.

**Database**	**Search string**
Scopus	TITLE (“Creative Thinking Ability”)

#### 2.3.2 Screening

The screening stage aimed to exclude articles irrelevant to the study (Ikram et al., 2021; Mehmood et al., 2022). Screening was guided primarily by subject area and document type. The authors focused exclusively on research articles within the fields of Social Science, Psychology, and Arts and Humanities. During this initial screening, studies were assessed using predetermined inclusion and exclusion criteria. Only peer-reviewed journal articles were retained, as they constitute primary sources of empirical evidence; conference papers, review articles, meta-analyses, books, book chapters, and series were excluded. No limits were imposed on the publication year to ensure wide temporal coverage. Following this process, 120 documents were removed (Table 2).

**Table 2:** The exclusion and inclusion criteria.

**Criterion**	**Eligibility**	**Exclusion**
Subject area	Social sciences, Psychology, Arts and humanities	Non-Social sciences, Psychology, Arts and humanities
Type of Literature	Journal (research articles)	Journals (re-view), books, book chapters, book series, conference proceedings

#### 2.3.3 Eligibility

**Figure 2:**
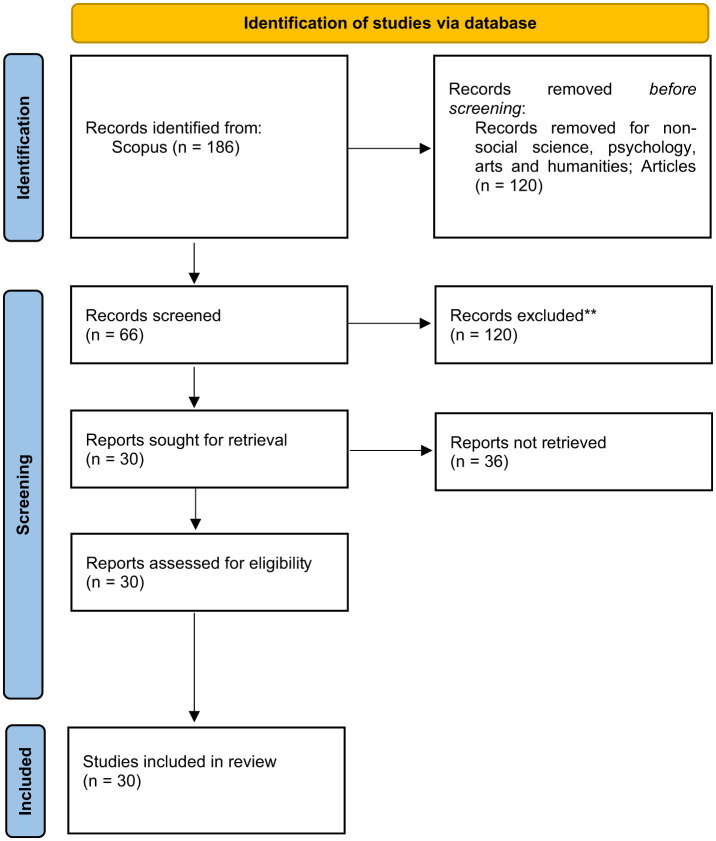
PRISMA Flow diagram. Source: PRISMA 2020 Flow diagram for systematic review.

In the third stage of the selection process, the remaining 66 studies underwent additional refinement. The authors closely reviewed the article titles, abstracts and full texts to confirm their alignment with the study's aims and criteria. After completing this eligibility assessment, 30 articles were deemed suitable and included in the final analysis (see Figure 2).

## 3 Results

### 3.1 The theory used by the reviewed articles

**Figure 3:**
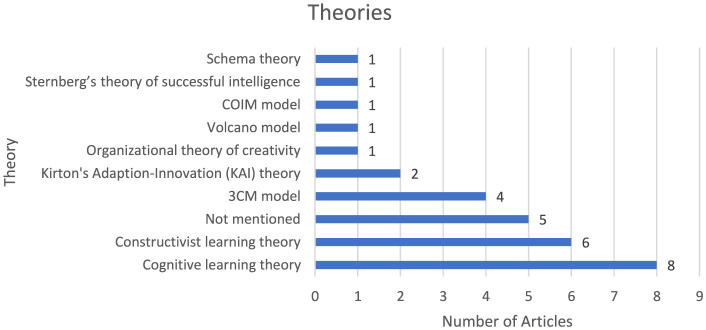
Distribution of theoretical frameworks across reviewed studies.

Figure 3 provides a summary of the theoretical frameworks cited in the reviewed studies. Of the total sample, 25 articles clearly articulated their theoretical underpinnings, whereas five did not specify any framework. Cognitive learning theory emerged as the most commonly utilized, appearing in eight studies. Other frequently adopted frameworks included constructivist learning theory and the 3CM model. Additional theories referenced were Kirton's Adaption-Innovation (KAI) theory, the Organizational Theory of Creativity, the Volcano Model, the COIM model, Sternberg's Theory of Successful Intelligence, and Schema Theory. Most of these were applied within the context of creative thinking ability research. Importantly, several studies adopted an integrative approach, drawing on multiple theoretical perspectives to support their analyses.

### 3.2 The method used by the reviewed articles

**Figure 4:**
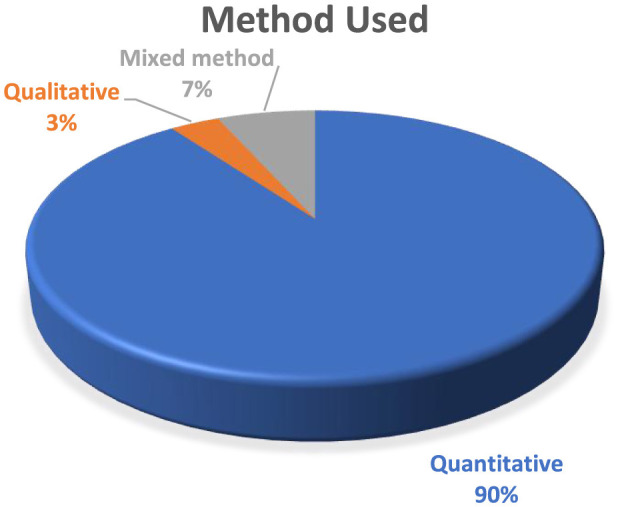
Distribution of research methods across reviewed studies.

Figure 4 depicts the distribution of research methods used in the reviewed articles. A dominant majority of studies twenty-seven papers (90%) adopted quantitative methods, while two studies (7%) utilized mixed methods. In contrast, only one study (3%) employed a qualitative approach. This strong inclination toward quantitative methodologies likely reflects the researchers' focus on examining the measurable effects of creative thinking abilities and their influence on student outcomes.

### 3.3 The research setting used by the reviewed articles

**Figure 5:**
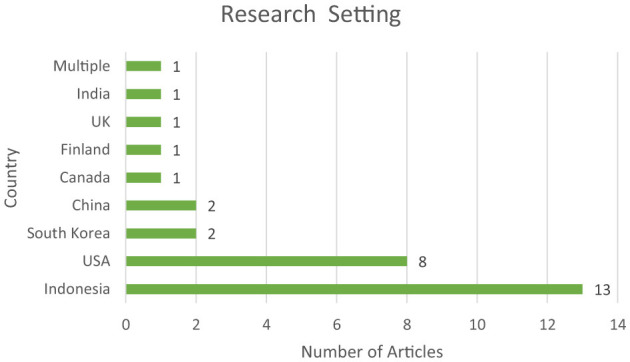
Distribution of research settings across reviewed studies.

Figure 5 presents the geographical distribution of the reviewed articles according to their countries of origin. The analysis showed that Indonesia contributed the highest number of articles, totaling 13 (43%) of the sample. The United States ranked second with eight articles (33%), while both South Korea and China each provided two articles (7%). Furthermore, individual contributions were noted from Canada, the United Kingdom, Finland, and India. This pattern underscores the predominance of research emerging from both Western and East Asian regions within the scope of this systematic literature review.

## 4 Discussion on research topics in students' creative thinking ability

### 4.1 Demographic influences

The reviewed studies indicate that demographic factors influence creative thinking, although their effects vary across contexts. Age-related differences suggest a general developmental trend, with older students often demonstrating stronger divergent thinking skills (Hong and Milgram, 2010; Lee, 2005), which aligns with cognitive development perspectives emphasizing increasing executive control and abstract reasoning over time. Gender-related findings are mixed. While some studies report higher verbal creativity among females (Torrance and Aliotti, 1969; Bart et al., 2015), others indicate context-dependent differences influenced by task type and assessment methods. Kaltsounis (1970) observed higher creative performance among deaf children, while Ogletree (1971) highlighted national and social-class differences in creativity levels. Collectively, these findings suggest that demographic variables interact with contextual and instructional factors, reinforcing socio-cultural and developmental psychology perspectives that view creativity as shaped by both individual and environmental influences.

### 4.2 Instructional approaches

Across studies, learner-centered instructional strategies consistently promote creative thinking more effectively than traditional approaches. Project-Based Learning (Setyarini et al., 2020; Ningsih et al., 2020), open-ended approaches (Fatah et al., 2016), the 3CM model (Wahyudi et al., 2021), and problem-solving strategies (Hendriana and Fadhilah, 2019) demonstrate positive effects across educational contexts. However, structured instructional models such as the 3CM framework (Wahyudi et al., 2021) and COIM module (Dewi and Mashami, 2019) yield more stable outcomes across creativity dimensions than less systematic interventions. This pattern suggests that instructional coherence and structural guidance are critical for sustaining creative development.

### 4.3 Assessment and measurement

Creativity assessment remains a contested area in the literature. The Torrance Tests of Creative Thinking (TTCT) continue to dominate creativity research due to their established use (Halpin and Halpin, 1973; Woodward and Sikes, 2015), yet concerns persist regarding domain specificity and scoring consistency. While some studies report acceptable psychometric properties (Rahayuningsih et al., 2021), others reveal inconsistencies in factor structures and interpretation (Wilson et al., 1954). These contradictions suggest that creativity assessment should adopt multi-method and context-sensitive approaches rather than relying solely on standardized instruments.

### 4.4 Cognitive and psychological factors

Internal psychological factors play a critical role in creative thinking development. Motivation and self-efficacy are strong predictors of creativity-related outcomes, particularly in domain-specific contexts (Halpin and Halpin, 1973; Rahyuningsih et al., 2022). Personality traits such as openness and schizotypy are also associated with higher creative performance (Poreh et al., 1993; Asquith et al., 2024), although their effects appear contingent on supportive learning environments. Cognitive processing abilities, including attention and information processing, further contribute to creativity (Mohanty, 2015). These findings reinforce cognitive and motivational models that view creativity as an interaction between cognitive resources, affective factors, and personality traits.

### 4.5 Technology integration

Technology-enhanced learning environments support creative thinking when aligned with pedagogical goals. Studies demonstrate that virtual reality enhances sensitivity and fluency (Hu et al., 2016) while ICT-based multimedia learning improves engagement and outcomes in STEM contexts (Mursid et al., 2022). Technology also supports dynamic student-teacher interaction and enhances the accessibility of digitalized instructional materials. The evolving role of technology in education not only enriches content delivery but also amplifies opportunities for creative expression and problem-solving. However, technology functions primarily as a mediating tool rather than an independent driver of creativity. This underscores the importance of pedagogical integration over technological novelty.

### 4.6 Creative thinking dimensions

Several studies focus specifically on the four core dimensions of creativity: fluency, flexibility, originality, and elaboration. The COIM module yielded significant gains across all four areas (Dewi and Mashami, 2019), while blended learning and the 3CM model improved these facets in mathematical contexts (Wahyudi et al., 2020; Wahyudi et al., 2021). Enhancements in these dimensions are often used as benchmarks for evaluating program effectiveness and personal growth (Noh, 2017). This highlights the need for instructional designs that equally support idea generation and refinement, consistent with multidimensional creativity frameworks.

## 5 Future direction of students' creative thinking ability research

### 5.1 Cross-cultural and contextual variations in creative thinking

A recurring suggestion is the need to extend research into diverse cultural, educational, and sociopolitical contexts. Studies emphasize the importance of moving beyond Western-centric models to explore how creativity develops across different cultures, school systems, and societal structures (Hong and Milgram, 2010; Bart et al., 2015; Ogletree, 1971). Researchers are called to investigate the influence of socio-cultural identity, such as sex-role identification (Torrance and Aliotti, 1969), social class, and regional educational policies on creativity. This line of inquiry will help dismantle one-size-fits-all assumptions in creativity assessment and pedagogy.

### 5.2 Refinement and diversification of assessment tools

Numerous studies advocate for deeper investigation into the tools and metrics used to assess creativity. Suggestions include testing alternative constructs like conceptual analysis and synthesis (Wilson et al., 1954), creating domain-sensitive instruments (Woodward and Sikes, 2015), and exploring ear preference and hemispheric asymmetry in schizotypal populations (Poreh et al., 1993). Calls for further replication and psychometric validation of creativity tools (Bart et al., 2015) also underscore a growing need to distinguish between different dimensions originality, fluency, flexibility and ensure their reliable measurement across populations and contexts.

### 5.3 Integration of advanced technology in instruction

There is a push toward exploring the role of immersive and digital environments in developing creativity. Virtual reality and digitalized instructional materials have shown promise in engaging students more deeply in creative thinking processes (Hu et al., 2016). Future research is encouraged to examine optimal instructional content design, the integration of these technologies across curricula, and the preparation of educators to deliver effective virtual experiences (Hu et al., 2016; Fatmawati, 2016). Investigating the technological augmentation of mind mapping, inquiry-based modules, and creative expression is also a suggested direction (Fatmawati, 2016; Dewi and Mashami, 2019).

### 5.4 Longitudinal and developmental perspectives

Several researchers highlight the need for longitudinal studies that trace the development of creative thinking across age groups and educational stages. Asquith et al. (2024) suggest exploring how openness and creative hobbies influence verbal creativity over time. Others call for developmental insights into how creativity unfolds in formative years and is shaped by early education, demographic factors, and evolving cognitive capacities (Hong and Milgram, 2010; Kaltsounis, 1970; Yamamoto, 1963). This theme highlights the value of mapping the trajectory of creativity rather than relying solely on cross-sectional snapshots.

### 5.5 Instructional innovation and differentiation

Researchers emphasize the need to explore new pedagogical models and evaluate their effectiveness across diverse educational contexts. Calls for further testing of models such as the Chemo-entrepreneurship Oriented Inquiry Module (Dewi and Mashami, 2019), blended Project-Based Learning (Mursid et al., 2022), and the 3CM model (Wahyudi et al., 2021) reflect a growing interest in differentiated, experiential learning approaches. Future studies could assess these models' impact across subject areas and demographics, paying attention to adaptability and long-term efficacy.

### 5.6 Cognitive, affective, and psychological variables

Future research should expand investigations into the affective and cognitive underpinnings of creativity. Many studies suggest exploring the roles of self-efficacy, motivation, attention, and personality traits like openness and schizotypy in shaping creative behaviors (Halpin and Halpin, 1973; Rahyuningsih et al., 2022; Mohanty, 2015; Asquith et al., 2024). Moreover, researchers advocate examining the interplay between cognitive novelty, framing, and creativity in mathematics and beyond. These directions could lead to richer theoretical models linking psychological traits with educational outcomes.

### 5.7 Inclusive education and underrepresented populations

There is growing recognition of the need for inclusive creativity research. Future directions call for examining underrepresented populations such as deaf students (Kaltsounis, 1970), schizotypal individuals (Poreh et al., 1993), and low-achieving students in blended learning environments (Mursid et al., 2022). These studies emphasize tailoring educational interventions to address the needs, abilities, and potentials of diverse learners rather than imposing uniform instructional models.

### 5.8 Institutional and environmental influence

Scholars recommend further exploration of how school types (e.g., Steiner vs. state schools), library settings, and other institutional environments affect creativity development (Ogletree, 1971; Noh, 2017). Recommendations include evaluating the operation and scalability of creative spaces such as “infinite imagination zones” and examining how institutional policies and physical environments support or hinder creative expression.

## 6 Conclusion and limitations

This systematic review critically examines the diverse research contexts and methodological approaches employed in investigating students' creative thinking ability. An analysis of 30 peer-reviewed articles reveals discernible trends and existing gaps within the literature, thereby offering substantive guidance for future scholarly inquiry. Theoretical underpinnings were found to be anchored predominantly in cognitive learning theory, constructivist learning theory, the 3CM model, and Kirton's Adaption-Innovation (KAI) theory. These frameworks underscore the conceptual importance of cognitive structures, instructional design, and learner adaptability in shaping creative thinking outcomes. Notably, such theories also emphasize the strategic role of knowledge systems and organizational resources in cultivating and sustaining creative capabilities.

The temporal and journal-wise distribution of the reviewed articles indicates a growing academic interest in this area. A significant methodological trend was the overwhelming reliance on quantitative approaches, with 90% of the studies employing this design. Only 7% of the studies adopted mixed-methods, and a mere 3% utilized qualitative methodologies, reflecting a strong preference for numerical data and statistical analysis to explore creative thinking constructs. Geographically, research output was concentrated in Indonesia (43%), followed by the United States, South Korea, and China, suggesting a heightened focus on creative thinking within Asian educational settings.

Overall, this review offers a consolidated perspective on the evolving field of student creative thinking ability. While the dominance of quantitative methods has facilitated generalizable insights, it also highlights a pressing need for methodological diversification, especially through qualitative and mixed approaches that better capture the nuanced, experiential dimensions of creativity. The geographic dispersion of research, particularly with emerging contributions from Asia and limited input from other global regions, affirms the international relevance of the topic yet exposes imbalances in representation that merit corrective attention.

Despite its contributions, several limitations must be acknowledged. A primary constraint lies in the restricted dataset limited to 30 articles retrieved exclusively from the Scopus database. Although the selected studies were rigorously evaluated, the exclusion of works from other repositories such as Web of Science or Google Scholar may have inadvertently narrowed the review's scope. Furthermore, relevant literature including dissertations, conference proceedings, regional journals, and non-English publications were omitted. This exclusion raises concerns about potential language and publication bias, which may have led to the underrepresentation of culturally diverse perspectives, particularly from non-Western education systems.

Another noteworthy limitation is the geographical concentration of studies, with a disproportionate focus on countries like Indonesia, the United States, South Korea, and China. The underrepresentation of regions such as Africa, South Asia, Latin America, and Eastern Europe constrains the generalizability of the findings. Given that creative thinking is influenced by numerous variables, such as demographics, instructional design, assessment practices, psychological traits, technological integration, and cognitive processes broader geographic inclusion is essential for developing globally applicable conclusions.

Additionally, the methodological homogeneity across the reviewed literature presents challenges. The dominance of quantitative methods, often reliant on self-report surveys and statistical testing, risks oversimplifying the multifaceted nature of creative thinking. Few studies employed qualitative or mixed-method designs, which are better equipped to explore learners' lived experiences, social interactions, and classroom dynamics. The lack of methodological variety thus limits the depth of understanding regarding how creative thinking manifests and develops in educational contexts.

An important omission in the review was the absence of a formal quality appraisal of the included studies. Without systematically evaluating the robustness of research designs, data collection tools, sample sizes, or analytical procedures, the reliability of the evidence base remains uncertain. Consequently, it is difficult to discern which studies offer the most credible and impactful findings.

Lastly, while this review usefully synthesizes thematic patterns such as demographic factors, instructional strategies, assessment methods, cognitive and emotional factors, technology use, and dimensions of creativity it may have overlooked more granular and context-specific variables that also shape students' creative development.

In conclusion, this literature review provides a valuable overview of current research on students' creative thinking ability. However, its contributions are tempered by limitations related to sample scope, regional bias, methodological uniformity, lack of quality assessment, and limited thematic granularity. Future research should strive to address these shortcomings by embracing more inclusive sampling, greater methodological rigor, and context-sensitive approaches to enrich our understanding of creative thinking in diverse student populations.

## 7 Implications

### 7.1 Theoretical implications

The findings of this review have several important theoretical implications for the study of creative thinking ability among students. First, the consistent use of cognitive and constructivist learning theories, along with models such as 3CM and Kirton's Adaption-Innovation theory, underscores their relevance in explaining the underlying mechanisms of creative development in educational settings. These theories emphasize the critical role of knowledge processing, learner adaptability, and instructional design in fostering creativity. However, the over-reliance on a limited set of frameworks suggests the need for broader theoretical development that incorporates additional constructs such as self-efficacy, emotional engagement and metacognitive awareness.

Additionally, the geographic concentration of research in countries such as Indonesia and the United States reveals a theoretical gap in understanding creativity from a global perspective. There is a pressing need to develop culturally inclusive frameworks that reflect the diverse educational realities of underrepresented regions like Africa, South Asia, and Latin America. Without such theoretical expansion, existing models may remain narrowly applicable and fail to account for cultural nuances in how creative thinking is expressed and nurtured.

Moreover, the review highlights the necessity for more integrative and multidimensional models of creativity. Current frameworks often treat creativity as a singular construct, whereas the research points to its complex, multifaceted nature encompassing cognitive, affective, social, and technological dimensions. Future theoretical work should aim to develop models that reflect this complexity. Finally, the limited use of qualitative methods in the reviewed studies points to a missed opportunity to refine the conceptualization of creative thinking through students' lived experiences. Incorporating qualitative perspectives could lead to more authentic and grounded theoretical understandings of how creativity develops within real-world learning environments.

### 7.2 Practical implications

From a practical standpoint, this review suggests several actionable recommendations for educators, policymakers, and curriculum designers. Most notably, the dominance of traditional quantitative approaches in literature reflects a broader trend in classrooms where instructional strategies often fail to capture the dynamic nature of creativity. Educators are encouraged to diversify their teaching methods by integrating strategies such as Project-Based Learning (PjBL), blended learning, inquiry-based models, and the 3CM approach. These pedagogies have shown effectiveness in enhancing students' creative thinking by fostering engagement, autonomy, and problem-solving.

Furthermore, the integration of technology in educational settings has emerged as a critical enabler of creativity. Tools such as virtual reality, interactive media, and digital content not only enhance instructional delivery but also stimulate students' imaginative capacities. Schools and institutions should invest in digital infrastructure and ensure that teachers are adequately trained to use technology in ways that support creative development. Professional development programs should thus be restructured to equip teachers with both theoretical knowledge and practical skills to cultivate creativity in diverse classroom contexts.

Assessment practices also require significant reform. The prevailing use of standardized, quantitative evaluations often overlooks the nuanced, divergent, and expressive qualities of creative thinking. As such, educators should adopt alternative assessment approaches, including portfolios, open-ended problem tasks, performance-based evaluations, and peer assessments. These methods can better capture students' originality, flexibility and fluency key dimensions of creativity.

Policy implications are also evident. Curriculum developers and educational leaders should recognize creative thinking not as a supplementary skill but as a core learning outcome across disciplines. This recognition necessitates the integration of creativity-focused objectives into national curricula, particularly within science, technology, engineering, mathematics (STEM), and language subjects. Finally, the geographical imbalance observed in the current research calls for greater international collaboration and cross-cultural research. Such efforts will not only diversify perspectives but also contribute to the development of globally relevant, context-sensitive strategies for fostering creative thinking among students worldwide.

## Data Availability

The original contributions presented in the study are included in the article/Supplementary material, further inquiries can be directed to the corresponding author.
