# Supplementary material for: Mapping the landscape of students' creative thinking ability: a systematic literature review and future pathways
Source: Front Psychol. 2026 Feb 11;16:1692056. doi: 10.3389/fpsyg.2025.1692056 (PMC12932539; doi:10.3389/fpsyg.2025.1692056)
Supplement: Supplementary file 1 [file Supplementary_file_1.docx]

Appendix A

**Detailed Overview of the included meta-analysis on Creative Thinking Ability**

| **Sr. No.** | **Author (Year)** | **Country** | **Method** | **Sample** | **Theoretical Framework** |
| --- | --- | --- | --- | --- | --- |
| 1 | Hong & Milgram (2010) | USA | Quantitative | 130 students (67M, 63F). | Not mentioned explicitly although Domain-general vs. domain-specific creative thinking debate; builds on Guilford (1950), Amabile (1996), and Baer (1998). |
| 2 | Wilson et al. (1954) | USA | Quantitative | 410 air cadets and student officers. | Cognitive learning theory. Hypothesising factors in creative thinking; identified factors including originality, redefinition, adaptive flexibility, spontaneous flexibility and sensitivity to problems. |
| 3 | Hu et al. (2016) | China | Quantitative | 104 university students from two classes - Experimental class (52 students) with Creative Thinking Instruction; Control class (52 students) with traditional instruction; 16 weeks, 3 hours/week. | Cognitive learning theory. |
| 4 | Lee (2005) | South Korea | Quantitative | 716 preschoolers aged 4-5 years from various city sizes: big cities (341), medium cities (240), small  cities (235). | Volcano Model for Creativity Measurement (inter-relates individual environment, creative thinking ability, creative personality, socio-cultural environment, and subject domain); influenced by Guilford (1950) and Torrance (1970, 1972). Cognitive learning theory. |
| 5 | Mursid et al. (2022) | Indonesia | Quantitative | 80 first- semester students from mechanical and automotive engineering education programs - Class A (40 students) and Class B (40 students); each class had 20 high and 20 low creative thinking ability students. | Constructivist learning theory. |
| 6 | Fatah et al. (2016) | Indonesia | Quantitative | Grade XI students from three senior high schools (high, middle and low categories); two classes per school (one experimental with open- ended approach, one control with conventional  teaching). | 3CM model. Creativity as high-order thinking skill; revision of Bloom’s taxonomy; mathematical creativity based on Poincare, Ervynck and Sriraman; self- esteem based on Coopersmith; open-ended approach based on Shimada and Becker. |
| 7 | Bart et al. (2015) | USA | Quantitative | 996 8th-grade students (503  boys, 493 girls, mean age 14.11) and  748 11th-grade students (407 boys, 341 girls, mean age 17.32) from a suburban independent public school district. | Kirton’s (1976) Adaption-Innovation (KAI) theory; Kim's (2006) factor structure of creativity model (fluency and originality = ‘innovative factor’; elaboration and abstractness = ‘adaptive factor’). |
| 8 | Poreh et al. (1993) | USA | Quantitative | Initial screening: 695 undergraduate students; Final sample: Schizotypal group (25 high scorers) and control group (60 low scorers); mean age 19.3 years, mean education 13.5 male students only. | Cognitive learning theory. |
| 9 | Rahayuningsih  et al. (2021) | Indonesia | Qualitative | Initial sample: 105 university students (seventh-semester students who completed Field Experience Program 1)  Interview sample: 4 students selected (2 from ‘very creative’ group with cognitive flexibility and fluency; 2 from ‘creative’ group with only cognitive flexibility). | Organisational theory of creativity.  Indicators of creative thinking based on Sriraman, Stenberg & Stenberg, and Singer & Voica; focused on cognitive flexibility and cognitive fluency; referenced Gestalt's creativity model for mathematicians' creative thinking process. |
| 10 | Nikkola et al. (2022) | Finland | Quantitative | N = 280 children   - Age: 13-83 months (mean = 65.74 months) - Gender: 138 boys, 138 girls - 29 children with special needs   From 23 municipal kindergartens/preschools. | Not mentioned |
| 11 | Halpin & Halpin (1973) | Canada | Quantitative | N = 62 undergraduate students   - Gender: 7 males, 55 females   Enrolled in educational psychology classes | Kirton’s KAI theory. |
| 12 | Yamamoto (1963) | USA | Quantitative | Teachers: N = 19 fifth-grade teachers   - 14 females, 12 married - Mean age: 35.4 years - 10 high creative, 9 low creative   Pupils: N = 461 fifth- graders   - 229 boys (49.7%) - Mean IQ: 116.71   124 high creative, 180 middle, 157 low creative | Cognitive learning theory. |
| 13 | Woodward & Sikes (2015) | USA | Quantitative | N = 75 undergraduate students   - 49 women, 26 men - Age: 18-25 years (mean = 20.16) - Musicians (n=42): 9 years music study   Nonmusicians (n=33): virtually no training | Cognitive learning theory. |
| 14 | TORRANCE  & ALIOTTI (1969) | USA | Quantitative | N = 118 fifth-grade pupils   - 59 girls, 59 boys - From three rural Wisconsin counties | Not mentioned |
| 15 | Setyarini et al. (2020) | Indonesia | Quantitative | Students from STIKOM Uyelindo Kupang   - Taking Statistics and probability courses - Experimental class (mean post-test = 78) - Control class (mean post-test = 70)   Specific sample N not provided | Cognitive learning theory. |
| 16 | Wahyudi (2020) | Indonesia | Quantitative | N = 27 students   - Primary School Teacher Education program, Class of 2017   Class A selected via random lottery from 7 classes and questionnaires | 3CM Model (Cool-Critical- Creative- Meaningful)  Contextual learning  Realistic mathematics  Meaningful learning (Brownell & Ausubel) |
| 17 | Dewi, C.A., Mashami (2019) | Indonesia | Quantitative | N = 20 students   - Chemistry Education Department students - Fall semester 2017- 2018   Saturated sampling (entire population included) | Chemo- Entrepreneurship Oriented Inquiry Module (COIM)  Inquiry-based learning processes  Creative thinking indicators: originality, fluency, flexibility, elaboration |
| 18 | Ogletree (2010) | ENGLAND, SCOTLAND, AND GERMANY | Quantitative | N = 1,165 children (grades 3-6)   - 666 state school, 499 Steiner school - 479 English, 193   Scottish, 493 German   - 557 boys, 608 girls   428 upper-middle, 584 middle, 153 lower class | No explicit framework  Contrasted educational approaches:  State schools: restrictive, academic  Steiner schools: less restrictive, art/motor-activity focused |
| 19 | Hendriana  (2019) | Indonesia | Quantitative | Two seventh-grade classes from SMP Negeri 1 Cilamaya Wetan • Class VII L (experimental) • Class VII I (control) • Randomly chosen classes • Junior high school students | No explicit theoretical framework Problem-solving approach as pedagogical strategy Emphasis on creative thinking for problem-solving |
| 20 | Noh (2017) | South Korea | Quantitative | N = 18 elementary school students (usable responses)   - Participated in library creative zone program for 6 months - Selected from elementary school near J library | Cognitive learning theory. |
| 21 | Ibrahim, Widodo (2020) | Indonesia | Quantitative | N = 72 students   - Random sampling technique - Divided into experimental and control groups   Students receiving advocacy approach vs. conventional learning | 3CM model |
| 22 | Fatmawati (2016) | Indonesia | Quantitative | N = 55 students   - Biology Education students - Enrolled in Biotechnology course - STKIP   Hamzanwadi Selong | Constructivist learning theory. |
| 23 | Rahyuningsih et al. (2022) | Indonesia | Mixed method | Sample details not fully specified   - Indonesian students   Focus on mathematical creative thinking and self-efficacy | 3CM model. |
| 24 | Mohanty (2015) | India | Quantitative | N = 80 children  • Classes 5 and 7  • From 2 residential/ashram schools (02)  • From 2 non-residential/formal schools (02)  • Medium of instruction: Bengali (mother- tongue)  • Individually administered tests | Sternberg’s theory of successful intelligence. |
| 25 | Ningsih et al. (2020) | Indonesia | Quantitative | N = 79 high school students  • Economic subject students  • West Bandung Regency, West Java  • Experimental class (Project-Based Learning)  • Control class (Expository methods). | Constructivist learning theory. |
| 26 | Kaltsounis (1970) | USA | Quantitative | (Grades 4, 5, 6)  • 67 deaf children (North Carolina  School for the Deaf)  • 351 hearing children (Suder Elementary School,  Jonesboro, Georgia)  • White children  • Hearing children from middle income families. | Constructivist learning theory. |
| 27 | Syutaridho et al. (2023) | Indonesia | Quantitative | Two classes of Grade VIII students  • Class VIII students at Lais State Middle School  • 2 classes selected via quota sampling  • Experimental group (PMRI approach)  • Control group (conventional learning). | Cognitive learning theory. |
| 28 | Wahyudi et al (2021) | Indonesia | Mixed method | Pre-service primary teachers  • Students from Faculty of Teacher Training and Education  • Primary school teacher education program. | Schema theory. |
| 29 | Asquith et al. (2024) | UK | Quantitative | 76 young people aged 14-20 years in three cohorts (14-15, 16-17, and 18-20 years). | Constructivist learning theory. |
| 30 | Lv et al. (2023) | China | Quantitative | 41 undergraduate civil engineering students (experimental group with ATDE mode vs. control group with traditional teaching). | Cognitive learning theory. |
